# Lower extremity compartment syndrome in the acute care surgery paradigm: safety lessons learned

**DOI:** 10.1186/1754-9493-3-11

**Published:** 2009-06-15

**Authors:** Jeffry L Kashuk, Ernest E Moore, Sarah Pinski, Jeffrey L Johnson, John B Moore, Steven Morgan, Clay C Cothren, Wade Smith

**Affiliations:** 1 Department of Surgery, Denver Health Medical Center and University of Colorado, Denver Health Sciences Center 777 Bannock St MC0206 Denver, Colorado 80204, USA; 2 Department of Orthopedic Surgery, Denver Health Medical Center and University of Colorado, Denver Health Sciences Center 777 Bannock St MC 0206 Denver, Colorado 80204, USA

## Abstract

**Background:**

Prompt diagnosis and decompression of acute lower extremity compartment syndrome (LECS) in the multisystem injured patient is essential to avoid the devastating complications of progressive tissue necrosis and amputation. Despite collaborative trauma and orthopedic management of these difficult cases, significant delays in diagnosis and treatment occur. Periodic system review of our trauma and orthopedic data for complications of LECS led us to hypothesize that delayed diagnosis and limb loss were potentially preventable events in our trauma center.

**Setting:**

Academic level 1 trauma center.

**Methods:**

We performed a prospective review of our trauma registry for all cases of LECS over a 7 year period (2/98–10/2005). Variables reviewed included demographics, injury patterns, tissue necrosis, amputation and mortality.

**Results:**

Eighty-three (10 female, 73 male) cases were reviewed. Mean age = 33.3 years (range 1–78). Mean ISS = 19.4, GCS = 12.5. Five (6.0%) had amputations; 7 (8.4%) died. Fractures occurred in 68.7% (n = 57), and vascular injuries were present in 38.6% (n = 32). In 7 patients (8.4%), a delayed compartment release resulted in muscle necrosis requiring multiple debridements, subsequent wound closure problems, and long term disability. Of note, none of these patients had prior compartment pressure measurements. Furthermore, 6 patients (7%) had superficial peroneal nerve transections as complications of their fasciotomy.

**Conclusion:**

In the multisystem injured patient, LECS remains a major diagnostic and treatment challenge with significant risks of limb loss as well as complications from decompressive fasciotomy. These data underscore the importance of routine surveillance for LECS. In addition, a thorough knowledge of regional anatomy is essential to avoid technical morbidity.

## Introduction

Establishing the diagnosis of lower extremity compartment syndrome (LECS) by physical examination in the multisystem injured patient is often difficult due to the presence of distracting injuries that often require urgent attention. Furthermore, associated head injury with depressed level of consciousness may significantly compromise the clinical examination and correlation with compartment pressure measurements. Additionally, the absence of lower extremity fractures may further reduce the index of suspicion and appreciation of subtle changes in compartment pressures. Lastly, the current acute care surgery paradigm suggests that the surgical hospitalist will likely be called upon to identify patients at risk for LECS and consequently must have an accurate management plan and be prepared for prompt surgical decompression when necessary.

A routine audit of the trauma data base at our Level 1 academic trauma center disclosed significant delays in diagnosis and treatment of LECS despite a multidisciplinary team of acute care specialists, including 6 trauma-acute care surgeons, 4 orthopedic trauma surgeons and a trauma- acute care surgery fellow. Accordingly, we hypothesized that delayed diagnosis and limb loss in lower extremity compartment syndrome was potentially preventable and sought to develop a management algorithm to improve our results.

## Methods

After appropriate IRB approval, a retrospective 7 year review of our prospective trauma database for lower extremity compartment syndrome was performed. All patients who developed lower extremity compartment syndrome during the course of their hospitalization in the presence of multi-system polytrauma were included. Variables evaluated included patient demographics (age, sex), ISS (Injury Severity Score), GCS (Glasgow coma score), as well as surgical intensive care unit and hospital length of stay. Issues evaluated in the diagnosis and treatment of compartment syndrome included injury pattern, whether fractures were present, associated vascular injuries, time from injury to diagnosis, documentation and frequency of compartment pressure measures, and technique of compartment pressure measurements. The method used for fasciotomy was documented, as well as the techniques of wound management till closure. The associated complications were divided into major (death, amputation, major muscle necrosis), and minor (wound issues from cellulitis to dehiscence necessitating delayed primary closure, skin grafting, and/or wound vac placement).

## Results

**Figure 1:**
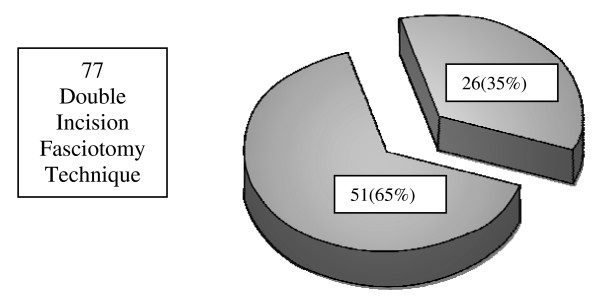
**Lower Extremity Compartment Syndrome Overall Complications: 51 (65%) Major/minor complications, 26 (35%) none**.

**Figure 2:**
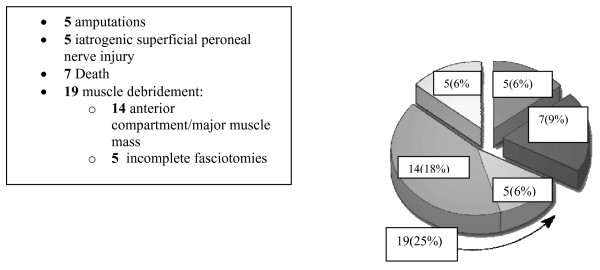
**Lower Extremity Compartment Syndrome-Major Complications**.

**Figure 3:**
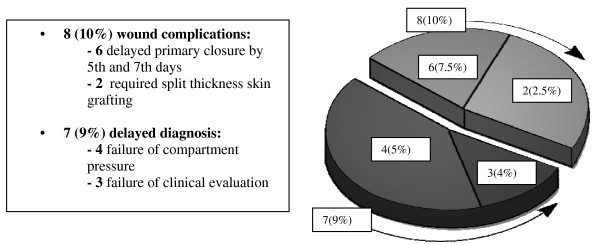
**Lower Extremity Compartment Syndrome-Minor Complications**.

Seventy-seven patients critically ill patients over 7 years underwent lower extremity fasciotomy using the double incision technique. Of note, mean GCS was 12.5 indicating that many patients were not able to fully cooperate for physical examination. The examination demonstrated fractures in 57 (68.7%) patients, and associated vascular injuries in 32 (38.6%) of the cohort. Overall, 51 (65%) patients developed complications. (Figure 1), with 36 (46%) considered major and 15 (19%) minor. Major complications (Figure 2) included 7 deaths, (all from associated multisystem trauma) 5 amputations, 5 iatrogenic superficial peroneal nerve injuries. Furthermore, 19 patients requiring muscle debridement, 5 of which were due to incomplete fasciotomies. Minor complications (Figure 3) included 8 wound issues ranging from mild cellulitis to complete dehiscence. This group included 6 patients who required delayed primary closure by the 5^th ^– 7^th ^days post-fasciotomy and 2 who required split-thickness skin grafting. Delayed diagnosis contributed to an additional 7 minor complications: 4 as a result of failure to measure compartment pressures and 3 as a result of delayed clinical evaluation for compartment syndrome

## Discussion

**Figure 4:**
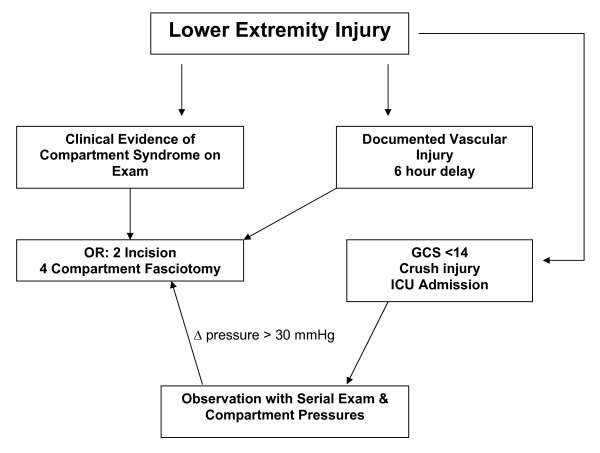
**Key Clinical Pathway (KCP) – Lower Extremity Compartment Syndrome**. Flow diagram for management of lower extremity compartment syndrome in patients with multi-system poly-trauma.

Despite newer diagnostic and wound care techniques, the recognition and treatment of compartment syndrome in the multisystem injured patient remains a diagnostic challenge. In our level 1 regional trauma center, despite an aggressive team of trauma-acute care surgeons and orthopedic trauma surgeons, we were surprised to note that our overall complication rate approached 65%. These results, (Figures 2 &3), included delayed diagnosis with associated muscle necrosis, amputation, nerve injuries, and a variety of wound closure issues. Given the fact that all patients in this series were polytrauma patients with multiple associated injuries, deaths occurred due to those injuries and were not specifically attributable to the lower extremity trauma discussed in this manuscript. We do believe, however, that patients with associated shock and hypotension due to their multisystem injuries are at significantly increased risk for compartment syndrome, and that in the face of severe torso, thoracic, or head injury, particular attention to lower extremity issues could be overlooked. Accordingly, we sought to develop a key clinical pathway (KCP) (Figure 4) to improve our results based upon improved diagnostic techniques, better documentation, thorough review and understanding of the regional anatomy, and meticulous surgical technique. These policies were further reinforced by multidisciplinary cooperation between the orthopedic service and the developing acute care surgery service. This review outlines the steps we took as our KCP evolved.

Physical examination remains the principle diagnostic technique in coherent trauma patients. The neurological examination of the lower extremity must include evaluation of the sciatic nerve and the peroneal and tibial divisions. The peroneal nerve courses around the neck of the fibula, which is a common site for its injury. The nerve supplies the muscles and tendons that allow dorsiflexion of the foot and toes, and eversion of the foot. The peroneal nerve innervates the extensor hallucis longus muscle, which supplies motor function to the great toe. Physical examination for sensory function of the peroneal nerve is accomplished by verifying sensation to the dorsal aspect of the foot, and the motor function of the nerve is assessed by asking the patient to dorsoflex the ankle and the great toe against resistance. The tibial nerve's sensory innervation can be verified by evaluating the muscles of the plantar aspect of the foot and the lateral aspect of the small toe. Motor function is verified by asking the patient to perform foot inversion and plantar flexion of the foot. The physical examination should be documented carefully so that the clinical course of potential compartment syndrome can be identified, which is critical to effective and timely decompression in the coherent patient. Cascio, et al [1] noted that documentation was often inadequate in many patients analyzed in an academic training program, with incomplete findings with regard to the presence of paresthesias, pain on passive stretch, sensory and motor deficits, as well as pain and tenseness. Although adjunctive use of compartment pressure measurements is intuitive, Ulmer [2] has noted that the sensitivity (positive predictive value) of clinical findings for diagnosis of compartment syndrome is low (13–19%), while the specificity and negative predictive value is high (97–98%), emphasizing that the clinical features of compartment syndrome of the lower leg are most useful by their absence in excluding the diagnosis.

Compartment pressure measurements are particularly useful in the obtunded patient where the physical exam is often unreliable. Lower leg compartment syndrome occurs when elevation of interstitial pressure in closed fascial compartments results in microvascular occlusion and interstitial pressure increase secondary to ischemia. This results in myoneural functional impairment and subsequent necrosis if not addressed in a timely fashion. Although it was recognized over 20 years ago that tissue necrosis may begin with pressures as low as 30 mm Hg, [3,4], others have suggested it requires higher pressures [5]. Olson [6] has proposed that the development of compartment syndrome depends not only on intra-compartmental pressures but also the systemic diastolic pressure, with improved accuracy when the delta pressure (systemic diastolic pressure-compartment pressure) exceeds 30 mmHg. Prayson, et al [7] described the baseline compartment pressure measurements in 19 isolated lower extremity fractures, noting that average compartment measurements were 35.5 ± 13.6 mm Hg in the injured leg versus 16.6 ± 7.5 mm Hg in the control leg. In a prospective analysis they concluded that use of direct compartment measurements with existing thresholds may not accurately establish the diagnosis of compartment syndrome, emphasizing the importance of improved accuracy of diagnostic ability along with clinical findings. Mcqueen, et al, [8] found in a prospective study of 116 patients with tibial fractures who underwent continuous monitoring of anterior compartment pressures for 24 hours that use of a delta pressure of 30 mm Hg as a threshold for fasciostomy led to no missed cases of acute compartment syndrome. The presence or absence of a tibial fracture in the extremity is a useful marker for potential compartment syndrome. Hope and McQueen [9] noted that the absence of a fracture led to significantly increased likelihood of muscle necrosis at the time of fasciotomy. On the other hand, in those patients with a fracture, 54% developed anterior compartment syndrome after reduction of their fracture, emphasizing the importance of early diagnosis and decompression in this group.

The currently accepted threshold for intervention to avoid necrosis is 6 hours of ischemia to the lower extremity, although correlation with the degree of compartment pressures is critical to identify when muscle compartment viability is lost [10-12]. Others [13] have suggested that patients with marginal compartment pressures may have salvageable limbs up to 12 hours, although debridement of muscle groups may be required. Unfortunately, many tertiary referral centers receive patients in transfer in whom significant delays may preclude timely fasciotomy for lower limp compartment syndrome. Finkelstein, et al, [14] found in a reterospective review that patients with significant delays had universally poor outcomes. One patient died of multiorgan failure and four others required lower limb amputation due to local infection and septicemia. Their experience, as well as others [15] emphasizes that fasciotomy in this group of patients converts a closed injury to an open one, and that compartment decompression is of no value once the delay results in irreversible ischemic changes. [16]

**Figure 5:**
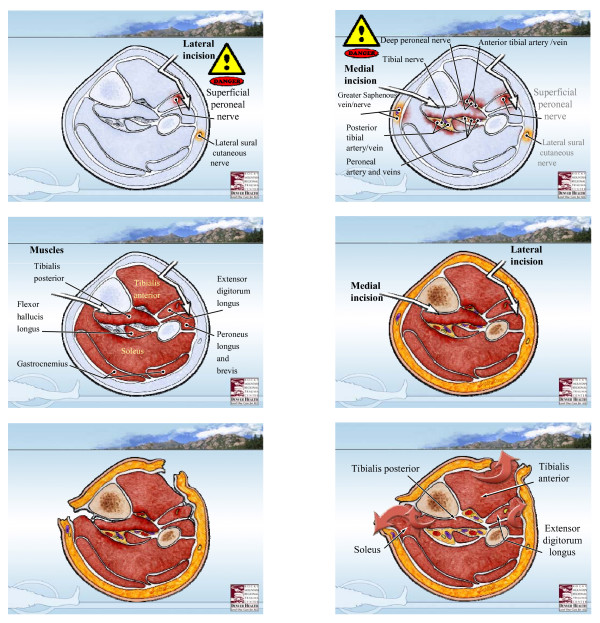
**Lower extremity four component fasciotomy-two incision technique**. Denver Health Medical Center's two-incision, four component fasciotomy technique for decompression of acute lower extremity compartment syndrome. (See text for details).

We believe that the most effective technique for decompression is the two incision- four compartment decompression technique as described by Mubarak and Owen [17] (Figure 5), and believe there is no role for the subcutaneous fasciotomy as it does not allow for adequate decompression[18,19]. The anterior and lateral compartments are approached thru a single longitudinal incision placed halfway down the leg halfway between the tibial crest and the fibula. This incision is located over the anterior intramuscular septum which separates the anterior and lateral compartments. This fascia is then opened proximally and distally with blunt pointed scissors, aiming for the patella proximally and distally to the center of the ankle. The lateral compartment is opened by directing the scissors towards the lateral malleolus, identifying the superficial peroneal nerve below which exits from the lateral compartment about 10 cm above the lateral maleolus. This compartment contains the peroneus brevis and longus muscles. The superficial peroneal nerve runs in the septum between the peronei and EDL. The anterior compartment contains the tibialis anterior, the EDL, EHL, and peroneus tertious muscles, the anterior tibial artery and deep peroneal nerve run deeply just anterior to the interosseous membrane, and can be identified between the tibialis anterior and EHL. The posteromedial incision is made to complete the decompression of the deep and superficial posterior compartments. This incision is about 15 cm long and courses approximately 2 cm posterior to the palpable edge of the tibia. Once one reaches the fascia, by undermining anteriorly to the posterior tibial margin, the saphenous vein and nerve as well as the sural nerve can be avoided by retracting anteriorly. Once the superficial posterior compartment is released, blunt tipped scissors are used to extend the fasciotomy proximally and distally. Finally, the deep posterior compartment is approached near where the soleus muscle originates from the proximal 1/3 of the tibia and fibula, and must be retracted to expose the fascia covering the FDL and the tibialis posterior. The neurovascular bundle of the posterior tibial artery and nerve are located between the tibialis posterior and the soleus, and should be carefully avoided during the release of this fascia. In so doing, the fascia is opened distally and proximally under the belly of the soleus.

We have employed a number of techniques to close fasciotomy wounds, including techniques such as shoelace/Jacob's ladder technique of graduated closure (dermatotraction) [20]. These techniques allow avoidance of cosmetically less acceptable skin grafting in most cases. A recent editorial emphasized the necessity for early diagnosis of acute compartment syndrome of the lower leg with fasciotomy, but also emphasized the importance of good surgical technique. [21]

Based upon our findings, we have made certain changes to our protocol to improve the accuracy of diagnosis of LECS and avoid technical complications in the operating room (Figure 5). Furthermore, we believe that the new acute care surgery paradigm [22,23] will result in increased responsibility for the care of these patients by the trauma-acute care surgeon. Accordingly, our program emphasizes a multidisciplinary approach to patients at risk for lower extremity compartment syndrome, allowing for improved understanding and knowledge of regional surgical anatomy in close cooperation with our orthopedic colleagues.

In summary, our experience suggests that a lower extremity compartment syndrome protocol, emphasizing careful physical examination, early aggressive fasciotomy when clinical indications exist, and liberal use of compartment pressure monitoring in situations where operative decompression may not be indicated or delayed, as well as thorough understanding of regional surgical anatomy, are the cornerstones of an educational program for optimal management of these patients.

## Competing interests

The authors declare that they have no competing interests.

## Authors' contributions

JBM, SP, and WS conceived the idea and obtained data for the study. JK and EEM drafted the manuscript. WS, SM, EEM, and JK critically reviewed the manuscript. Final revisions were made by JK and EEM. All authors contributed patients to this study, and all authors read and approved the final version of this manuscript.
